# Modulating the Gut–Muscle Axis: Increasing SCFA-Producing Gut Microbiota Commensals and Decreasing Endotoxin Production to Mitigate Cancer Cachexia

**DOI:** 10.3390/microorganisms13061356

**Published:** 2025-06-11

**Authors:** Sagnik Roy, Amir Hossein Alizadeh Bahmani, Mark Davids, Hilde Herrema, Max Nieuwdorp

**Affiliations:** 1 Department of Vascular Medicine, Amsterdam UMC location University of Amsterdam, Meibergdreef 9, 1105 AZ Amsterdam, The Netherlands; s.roy@amsterdamumc.nl (S.R.); a.h.alizadehbahmani@amsterdamumc.nl (A.H.A.B.); m.davids@amsterdamumc.nl (M.D.); h.j.herrema@amsterdamumc.nl (H.H.); 2 Department of Experimental Vascular Medicine, Amsterdam UMC Location University of Amsterdam, Meibergdreef 9, 1105 AZ Amsterdam, The Netherlands

**Keywords:** cancer cachexia, gut microbiota, dysbiosis, short-chain fatty acids (SCFAs), branched chain amino acids (BCAAs), bile acids, intestinal barrier integrity, inflammation, microbiome-based therapies, probiotics, prebiotics, synbiotics, fecal microbiota transplantation

## Abstract

Cancer cachexia is a multi-organ and multifactorial syndrome characterized by muscle wasting (with or without adipose tissue loss) and systemic inflammation in patients with advanced malignancies. Gut microbiota dysbiosis, particularly the depletion of short-chain fatty acid (SCFA)-producing bacteria, may contribute to the progression of cancer cachexia. Studies in both murine models and humans consistently associate cachexia with a decline in SCFA-producing gut microbiota commensals and an overgrowth of pro-inflammatory pathobionts. These microbial imbalances may lead to reduced levels of SCFAs and branched-chain amino acids (BCAAs) and alter the normal bile acid profile. BCAAs and the maintenance of a normal bile acid profile are associated with muscle synthesis and decreased breakdown. While SCFAs (acetate, propionate, and butyrate), contribute to intestinal barrier integrity and immune regulation. SCFA depletion may increase gut permeability, allowing bacterial endotoxins, such as lipopolysaccharide (LPS), to enter the bloodstream. This may lead to chronic inflammation, muscle catabolism, and impairment of anabolic pathways. Interventions targeting gut microbiota in preclinical models have mitigated inflammation and muscle loss. While clinical data are limited, it suggests an improvement in immune functions and better tolerance to anticancer therapies. Current evidence is predominantly derived from cross-sectional studies suggesting associations without causality. Thus, future longitudinal studies are needed to identify biomarkers and optimize personalized therapy.

## 1. Introduction

**Figure 1:**
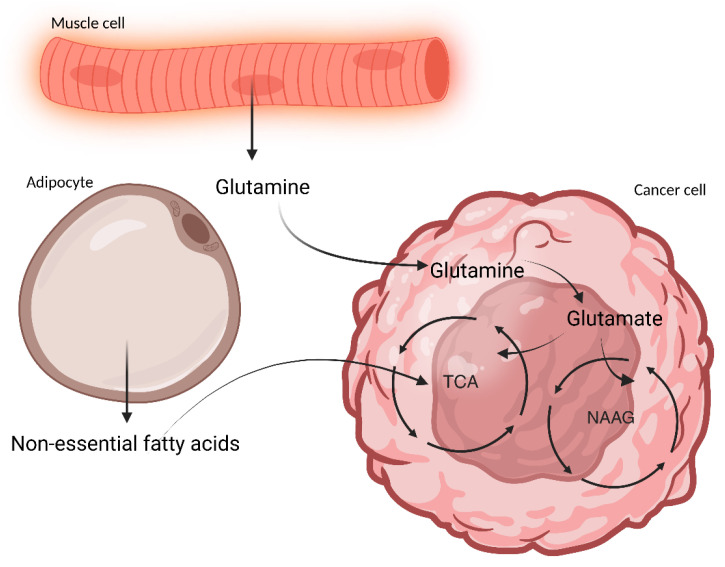
Cachexia feeds tumor progression. Muscle atrophy releases glutamine in the blood stream which is taken up the energy-demanding cancer cell where it is converted to glutamate. This is then further converted to α-ketoglutarate to feed the tricarboxylic acid cycle (TCA cycle) resulting in energy production for tumor cells in the form of ATP (adenosine triphosphate) [1]. Glutamate is also stored as N-acetyl-aspartyl-glutamate (NAAG) via the NAAG cycle and found in high levels in advanced-stage cancer [11]. Along with skeletal muscle degradation, adipose tissue degradation contributes to cancer progression via lipolytic breakdown of triacylglycerols producing non-essential fatty acids (NEFAs) used by cancer cells in TCA cycle, thereby maintaining its high energy demand and, as a result, proliferates [1]. Created using BioRender.com.

Cancer cachexia is a multifactorial syndrome in patients with a malignancy and characterized by unintentional weight loss, skeletal muscle loss (with or without loss of adipose tissue mass), and systemic inflammation [1,2]. About 3/4 of pancreatic and gastric cancer patients in advanced-stage are affected by cachexia and it has been shown to contribute to 1/5 to 1/3 of cancer-related deaths [3,4]. Cachexia leads to progressive functional impairment and poor tolerance to anticancer treatments, which leads to diminished quality of life and, thereby, reduced survival [5,6,7]. It has also been linked to gut barrier dysfunction, impaired insulin sensitivity and food intake dysregulation [1,2], along with multi-organ wasting including liver, brain, bone, and heart [8]. Cachexia has thus been described as a metabolic disorder marked by tissue wasting, overall catabolic state, and anabolic signal resistance which releases nutrients in the blood stream, feeding energy-demanding highly proliferative cancer cells [1,9,10]. Figure 1 illustrates that muscle and adipose cell wastage provides glutamine and non-essential fatty acids that feed cancer cells via the Krebs cycle (tricarboxylic acid cycle) [1,11].

In this regard CASCO, a tool developed by Argiles et al. has been validated on 169 cancer patients by Busquets et al. as a tool to identify pre-cachectic patients as well as to stage cachexia according to inflammation/immune suppression/metabolic disturbances, weight loss and body composition, physical performance, quality of life, and anorexia [12,13]. It classifies cachexia-positive individuals into mild (<25), moderate (26–50), severe (51–75), and terminal (76–100) cachexia on a scale of 0 to 100 [13].

Traditional interventions like nutritional support [14,15], appetite stimulants [16,17,18,19], and anabolic agents [20,21] have shown limited success in reversing cancer cachexia. This highlights a critical need to look for novel pathways underlying its pathogenesis [22]. Recent studies suggesting the gut microbiota’s role in host metabolism, immunity, and inflammation connects it to cancer cachexia pathogenesis pathways [23,24,25]. The diverse community of microbes present in the whole human gastrointestinal (GI) tract interacts with the immune system by influencing energy harvest from diet and producing bioactive metabolites. Increasing evidence in metabolic disorders indicates that commensal bacteria may help maintain metabolic homeostasis, intestinal barrier integrity, and immune tolerance in healthy individuals [26,27,28]. In cancer patients, this microbial equilibrium may be disrupted (“dysbiosis”) independent of chemotherapy or anorexia [26]. It potentially contributes to cachexia by promoting systemic inflammation [29], catabolic processes [30], and altering amino acid availability [31]. Notably, preclinical models show that germ-free (GF) compared to pathogen-free (PF) mice exhibit reduced muscle mass and increased muscle atrophy markers (Atrogin-1, MuRF-1, and FoxO) [32], characterized by reduced insulin-like growth factor (IGF-1) expression, altered amino acid metabolism (elevated glycine and alanine, reduced choline), and impaired neuromuscular signaling (decreased Rapsyn and Lrp4) [32]. Moreover, fecal microbiota transplantation (FMT) from PF mice or SCFA treatment partially restores the PF phenotype in GF mice [32]. Similarly, antibiotic-mediated (cocktail of ampicillin, vancomycin, neomycin, metronidazole, and amphotericin) microbiota depletion in healthy mice induced muscle wasting, likely via aberrant dietary nutrient uptake as well as altered production of gut microbial metabolites (e.g., bile acids) affecting muscle metabolism [33]. Thus, we see a potential “gut–muscle axis” appear where muscle protein metabolism and inflammation are modulated by gut microbiota and their metabolites.

Short-chain fatty acids (SCFAs), such as acetate, butyrate, and propionate formed from fermentation of dietary fiber by gut microbes representing a major carbon flux to the host [27], are key metabolites that may mediate the gut–muscle axis. SCFA butyrate improves intestinal integrity by strengthening intestinal epithelial tight junction while also acting as the primary energy source for colonic epithelial cells [34]. Whereas SCFAs acetate and propionate can influence gluconeogenesis, lipid metabolism, and appetite signaling [27,35]. SCFAs exert anti-inflammatory effects by binding to G-protein coupled receptors (e.g., GPR41/43) on immune cells and inhibiting histone deacetylases, leading to cytokine modulation [36]. Cancer cachexia is associated with impairment of gut barrier function and systemic inflammation. Its changes have been correlated to reduced fiber intake [37] and reduction of SCFA-producing microbes [38]. A study on a murine cachectic tumor model found reduced levels of acetate and butyrate in the lower cecal region [38]. Similarly, clinical research also found reduced levels of fecal acetate in cachexia-positive cancer patients [39]. This suggests SCFA deficiency could be a link between microbiota alterations and the pro-inflammatory and catabolic state seen in cachexia.

**Figure 2:**
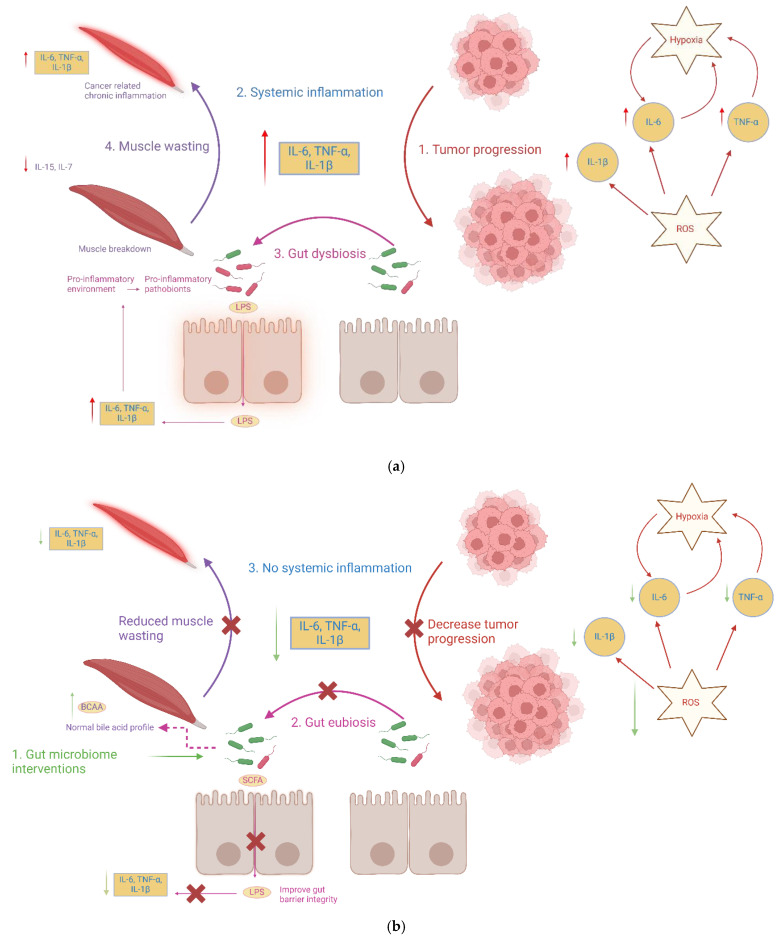
Gut microbiota interventions is a multi-modal approach focusing on mitigating multi-organ and multifactorial syndrome cancer cachexia. (**a**) Tumor progression increases pro-inflammatory cytokines (IL-6, IL-1β, and TNF-α) which fuels systemic inflammation cycle. Systemic inflammation further affects the gut by creating a pro-inflammatory environment supporting pro-inflammatory pathogens, thereby increasing LPS (lipopolysaccharide) production and decreasing the production of SCFAs (short-chain fatty acids). It prevents the energy supply to colonocytes, thereby damaging the gut barrier integrity, resulting in the LPS passing through the gut barrier. It again fuels the systemic inflammation, maintaining a chronic state of skeletal muscle inflammation, thereby increasing muscle atrophy gene expression. Hence, resulting in muscle wasting which again fuels the systemic inflammation cycle increasing tumor progression and the vicious cycle continues. (**b**) Gut-microbiota interventions can potentially “reset” gut microbiota towards that of a healthier, non-cachectic state. By enriching SCFA-producing commensals, preventing loss of BCAAs, and maintaining bile acid homeostasis which is generally marked by an increase of secondary bile acids (microbial metabolites) production. These changes bring the gut into an “eubiotic state”, which may break the vicious cycle of systemic inflammation, potentially reducing skeletal muscle wasting and tumor progression. Color and arrow legend: Red arrows indicate pro-inflammatory, pro-cachectic, or tumor-promoting pathways. Green arrows indicate therapeutic effects mediated by gut microbiome interventions. Purple arrows represent gut–muscle axis communication mediated by microbial dysbiosis and metabolites (e.g., SCFAs, LPS). Dashed arrows indicate indirect or modulatory effects (e.g., BCAA metabolism, bile acid modulation). Yellow boxes denote pro-inflammatory cytokines (IL-6, IL-1β, TNF-α). Red tumor cells represent tumor burden. Maroon muscle fibers depict skeletal muscle undergoing wasting or recovery. Pink epithelial cells represent gut barrier integrity status. Green and red bacteria represent commensals and pathobionts, respectively. Orange border highlights compromised gut permeability and LPS translocation. Figure created with BioRender.com.

Another factor connecting gut microbes to cachexia is gut barrier integrity. The intestinal mucosal barrier normally prevents the translocation of bacteria and endotoxins (such as lipopolysaccharide, LPS) into circulation. Dysbiosis can undermine this barrier by reducing levels of butyrate-producers (which support the epithelial barrier) and by enriching pathobionts that trigger mucosal inflammation [40]. In a mouse model of cancer cachexia (Apc^Min/+^ colorectal cancer model), increased intestinal permeability was observed with cachexia onset; this leakiness was associated with elevated circulating LPS and interleukin (IL)-6 and marked activation of inflammatory pathways [41]. Similarly, cachectic mice bearing colon tumors showed higher expression of gut epithelial claudins (indicating disrupted tight junction regulation) and evidence of bacterial translocation with elevated serum LPS-binding protein (LBP) [26]. Translocated gut microbial products can stimulate Toll-like receptors and immune cells systemically, fueling a chronic inflammatory state that promotes muscle proteolysis and metabolic derangements [42]. Human studies mirror these findings: cachectic patients with gastrointestinal cancers exhibited increased intestinal permeability and tight junction abnormalities [43]. The same study also showed that cachexia-positive patients had a higher endotoxin load and inflammatory cytokines (IL-6, TNF-α, IFN-γ) compared to non-cachectic patients [43]. This paints a picture wherein tumor-driven inflammation and gut microbiota changes work together to damage the gut barrier, leading to endotoxemia that further induces systemic inflammation. Figure 2a shows the vicious cycle of systemic inflammation that underlies cancer cachexia pathogenesis.

Given the potential role of the gut microbiome in modulating inflammation, metabolism, and immunity, investigating microbiota alterations in cancer cachexia may have clinical implications. A better understanding of the role of the gut microbiome and its produced metabolites and their interactions with the immune system can help to identify novel biomarkers for early cachexia diagnosis and to uncover potential therapeutic targets. Discovering these underlying mechanisms opens the door to microbiome-focused interventions, alongside conventional cachexia treatments [44]. This review provides a comprehensive overview of current knowledge on gut microbiota, metabolites such as SCFAs, gut barrier dysfunction, and inflammation in cancer cachexia. In addition, it compares findings across different tumor types (e.g., lung, colon, leukemia) and discusses emerging therapeutic strategies to modulate the microbiota–gut–muscle axis.

## 2. Gut Microbiota Targeted Intervention May Mitigate Cancer Cachexia Linked Gut Dysbiosis, Muscle Wasting, and Systemic Inflammation

### 2.1. Cancer Cachexia Is Linked to Gut Dysbiosis Marked by a Loss of SCFA-Producing Gut Microbiota Commensals and Enrichment of Pro-Inflammatory Pathobionts

Microbial diversity: Many studies indicate that cancer cachexia is associated with shifts in gut microbiota diversity and composition. However, results on overall diversity have varied. In two murine models of cachexia, a leukemia model (Ba/F3 model transfected with Bcr-Abl) [45] and a colorectal cancer model (C26 colon carcinoma) [46,47], amplification of the V5-V6 region of 16S rRNA from cecal microbiota analysis reveals significant reductions in α-diversity (species richness or Shannon index) with an affected 25 and 54 taxa, respectively, observed in cachectic mice compared to non-tumor controls, suggesting that the cachectic state favors a less diverse microbial community [48]. In the same study, OTU level Morisita-Horn beta diversity showed 74% and 95% variation in BaF and C26 models, respectively, compared to the no-tumor control [48]. Contrastingly, a study of cachectic mice bearing Lewis lung carcinoma (LLC) found an increase in gut microbial richness, while there was no variation in evenness (Simpson’s index) from the stool sample-amplified V4-V5 region of 16S rRNA compared to saline-injected controls [49]. Meanwhile, NMDS (non-metric multi-dimensional scaling) beta diversity (microbial community structure dissimilarity) showed a slight differentiation of 12.7% between the groups [49]. The LLC model is an established model of cancer cachexia, but this finding of increased diversity is unusual and opposite to what was seen in the leukemia and colon cancer models. The reasons for this discrepancy are still unclear; it may relate to differences in tumor type, different 16S rRNA region or sample type. Another LLC murine model study comparing cancer cachectic and no-tumor controls using V3-V4 16S rRNA sequencing found that the Shannon index (evenness) was significantly higher in the control groups. However, the difference in evenness was only slightly higher despite being statistically significant [50]. The same study also showed a distinct cluster between cancer cachectic vs. no-tumor control by Bray–Curtis dissimilarity evaluated using principal coordinate analysis (PCoA) [50]. In human studies, differences in diversity have been subtle. A clinical case-control study on 107 patients with various cancers (27 pancreatic, 4 ovarian, 52 breast, and 24 lung) with 33 being cachectic (18 pancreatic, 1 ovarian, 9 breast, and 5 lung) and 76 healthy controls (patients’ household partners or relatives) found no significant change in fecal microbiota (amplified from V4 region of 16S rRNA) α-diversity (species richness and Shannon index) between cachectic cancer patients and non-cachectic or healthy controls [39]. Similarly, NMDS β-diversity showed no clear clustering while the dendrogram only trended toward separation by cachexia and BMI status [39]. Another human study by Wu et al. compared chemotherapy-induced cachexia in patients with gastric cancer (28 cachectic, 18 non-cachectic) compared to 30 healthy controls from the same region, as well as at different timepoints before and after chemotherapy treatment [51]; 16S rRNA sequencing by amplifying the V3-V4 region from the fecal samples showed a higher level of diversity and unevenness compared to controls while post-chemotherapy showed decreased richness and Chao1 index compared to the pre-chemotherapy state [51]. Beta-diversity using partial least squares discrimination analysis (PLS-DA), showed distinct clustering of cancer patients compared to controls [51]. Although the data are limited and contrasting, it might suggest that while certain preclinical models show a collapse of microbial diversity in cachexia, in human cancer patients, the overall diversity reduction may be mild or confounded by factors like anorexia [48], tumor type, diet, or (chemotherapy and checkpoint inhibitor) treatment. Thus, changes in specific taxa abundance (composition) may be more informative than global diversity indices in linking the microbiome to cachexia.

Taxonomic composition changes: Despite some heterogeneity, there are recurring patterns of dysbiosis associated with cancer cachexia across studies. A common observation is the depletion of commensal genera that are regarded as typically beneficial (including SCFA-producing bacteria) and an over-representation of bacteria with pro-inflammatory or opportunistic characteristics. For example, in cachectic mice with colon tumors (C26 model), 16S rRNA-amplified V5-V6 region sequencing from the cecal area revealed a bloom of the family Enterobacteriaceae (phylum Proteobacteria) coinciding with a marked reduction in butyrate-producing *Clostridia* (families Ruminococcaceae and Lachnospiraceae) [40]. The findings were supported by a previous study done by Byndloss et al. showing streptomycin treatment in mice results in a reduction of butyrate-producing microbes, leading to an increase in nitric oxide in the cecal area, thereby supporting *E. coli* (an Enterobacteriaceae member) growth by acting as their respiratory electron acceptor [52]. Specifically, *Klebsiella oxytoca* (also an Enterobacteriaceae member) significantly overgrew in cachectic C26 mice at the expense of the normally dominant anaerobic Firmicutes; experimentally adding *K. oxytoca* to mice was sufficient to induce gut barrier dysfunction and local inflammation in cachectic (but not healthy) hosts [40]. Similarly, Bindels et al. noted that cachectic mice (both leukemia and colon models) had increased Enterobacteriaceae and increased Bacteroidetes (e.g., genus *Parabacteroides*), coupled with a depletion of *Lactobacillaceae*/*Lactobacillus* and reduced Clostridiales (Firmicutes) [48]. Specifically, both cancer cachectic models BaF and C26 showed a four-fold decrease in *Lactobacillus johnsonii*/*gasseri*, with a 2698-fold and 4676-fold increase in *Escherichia* in BaF and C26, respectively [48]. An increase in Enterobacteriaceae in the gut was the most prominent microbial signature in both models, and it was also found in later studies of different cancer cachexia models. In the LLC lung cancer cachexia model, the dysbiosis pattern diverged from the gastrointestinal tumor models. De Maria et al. found that LLC-bearing cachectic mice had an increase in Firmicutes (particularly classes Bacilli and Clostridia, including families *Staphylococcaceae*, *Turicibacteraceae*, *Ruminococcaceae*, *Lachnospiraceae*) and also higher relative abundance of Cyanobacteria and Tenericutes, compared to controls. Conversely, certain Bacteroidetes taxa were decreased in LLC cachexia (families *Prevotellaceae* and *Bacteroidaceae*, and species *Bacteroides acidifaciens* were lower in cachectic vs. non-tumor mice) [49]. Among Proteobacteria in the LLC model, there was a shift in composition rather than an increase: Betaproteobacteria and Deltaproteobacteria expanded, while Gammaproteobacteria overall decreased except for the Enterobacteriaceae (e.g., *Escherichia coli* was specifically increased) [49]. These findings indicate that different tumor types can imprint distinct signatures on the gut microbiome during cachexia. Similar to the LLC model by de Maria et al., Feng et al. also found an increased abundance of Firmicutes in C26 mice after sequencing of V3–V4 16S rRNA [53]. However, the previous study of cachexia showed taxonomic alteration between cachexia induced by intra-abdominal tumors differs from extra-abdominal tumors. But the actual scenario seems to involve an overgrowth of Gram-negative Enterobacteriaceae and alteration of anaerobic Firmicutes rather than a flat decrease (decrease in Lachnospiraceae and increase in Eubacteriaceae in C26 mice from V3-V4 region of 16S rRNA amplified) [53]. It is worth noting, however, that despite these differences, both patterns involve an imbalance that could promote inflammation (either via Gram-negative bacteria overgrowth producing endotoxins like LPS, or via other pathobionts blooming). A study by Jabes et al. focusing on mycobiota alterations in the LLC model of CC [54,55] found fungal dysbioisis associated with the LLC-CC model presents features observed both in obesity (reduced proportion of Mucoromycota) and CRC/ME/IBD (increased proportions of Sordariomycetes, Saccharomycetaceae, and Malassezia) [56]. This mycobiota alteration remains to be verified in other CC model and/or whether they are specific to LLC-CC model [56].

Human studies of cancer cachexia echo some of these taxonomic changes found in animal models of CC. In a cohort of 33 cachectic cancer patients (mixed tumor types) versus 74 non-cachectic patients and 76 healthy controls (household partners with similar diet and environmental conditions), Proteobacteria were significantly more abundant in cachectic individuals [39]. This enrichment was driven largely by an unclassified genus of Enterobacteriaceae that was highly elevated [39]. The genus *Veillonella* (a lactate-fermenting anaerobe often linked to pro-inflammatory states) was also markedly increased in cachectic patients [39]. Meanwhile, taxa from the Firmicutes phylum were under-represented in cachexia: for instance, genera from the Lachnospiraceae and Ruminococcaceae families (important butyrate producers) tended to be lower [40,53]. *Megamonas* and *Peptococcus* (Firmicutes genera) were significantly reduced compared to non-cachectic patients, consistent with prior mouse studies showing alteration within Firmicutes in cachectic hosts [39,40].

Although specific bacterial/fungal shifts can vary by cancer type, a recurring theme is dysbiosis, characterized by loss of beneficial SCFA-producing commensals and enrichment of potential pathogens or pro-inflammatory microbes. Table 1 illustrates the dysbiosis signature for several representative studies.

### 2.2. Cancer Cachexia-Linked Dysbiosis Depletes SCFAs, BCAAs, and Alters the Bile Acids Profile

Cachexia-associated dysbiosis is associated with changes in gut microbial metabolites, especially short chain fatty acids (SCFAs), showing significant alterations. In the mouse models of cachexia, a loss of key fiber-fermenting taxa (e.g., Lachnospiraceae) was accompanied by decreased cecal SCFA concentrations. In line with these results, Potgens et al. reported that cachectic C26 tumor-bearing mice were characterized by significantly lower cecal butyrate and acetate compared to non-tumor controls [38]. This SCFA drop was temporally aligned with the expansion of Enterobacteriaceae and reduction of Ruminococcaceae and Lachnospiraceae (butyrate-producers) in those mice, which was also strongly correlated with increased amino acids found in the gut (also increased in liver and decreased in plasma) [40]. Similarly, another study in C26 cachectic mice showed a significant decrease in Lachnospiraceae and corresponding reduction in total SCFAs, confirming that fiber-degrading, butyrate-producing microbes are diminished in cachexia [53]. Interestingly, that study also found alterations in bile acid metabolism (with reduced microbial secondary bile acids), indicating broader metabolic dysregulation linked to the microbiota in cachexia [53].

Human data, while limited, are consistent with intestinal SCFA depletion in cachexia. Total fecal SCFA levels tended to be lower in cachectic cancer patients compared to non-cachectic patients and healthy controls [39]. The median total SCFA concentration in feces of cachectic patients was ~38.6 mM/g versus 48.8 mM/g in non-cachectic cancer patients (a ~20% reduction) [39,40]. When broken down by individual SCFAs, acetate was significantly reduced in cachectic patients (*p* < 0.05 compared to the non-cachectic group) [39]. Fecal acetate in cachectic patients was about 30% lower than in non-cachectic patients [39]. Propionate and butyrate were also lower on average in cachexia, though these differences did not reach statistical significance in that cohort [39]. These human findings parallel earlier mouse results where acetate and butyrate drops were noted in cachexia [38]. It is notable that acetate was the most significantly affected SCFA in the human study [39]. Acetate is the most abundant SCFA and plays roles in appetite regulation [63], lipogenesis [64], and gut immune homeostasis [65]. Lower acetate in cachectic patients could reflect both reduced microbial production (due to dysbiosis) and increased utilization or absorption. Acetate has been linked to body weight regulation and insulin sensitivity, and in animal models, it can modulate muscle and adipose tissue metabolism [65]. Thus, a deficiency in acetate (and other SCFAs) in cachexia may conceivably contribute to the negative energy balance and muscle catabolism characteristic of the syndrome.

Despite the clear anti-inflammatory and barrier-supporting functions of SCFAs, direct correlations in cachectic patients have been hard to demonstrate. This is likely due to complex confounding factors and technical limitations associated with measurement of plasma SCFA levels. In the human study, fecal SCFA concentrations did not significantly correlate with C-reactive protein (CRP), leukocyte count, or fecal calprotectin [66]. One might expect SCFA depletion to associate with higher gut inflammation (since SCFAs normally suppress inflammation), but in that study, most patients (cachectic and non-cachectic alike) had elevated CRP due to cancer, possibly masking any difference [66]. Nonetheless, preclinical evidence strongly suggests SCFA loss is functionally important. The impaired gut barrier in cachectic mice is thought to be partly due to reduced butyrate availability for colonocytes [38]. Butyrate normally induces mucin and tight junction protein expression, maintaining barrier integrity [27]. Its depletion in cachexia could lead to weakened tight junctions and easier bacterial translocation. Acetate and propionate can also modulate immune responses; their reduction may tilt the balance toward a pro-inflammatory state [36]. Moreover, SCFAs are known to influence host metabolism: propionate serves as a gluconeogenic substrate in the liver, and acetate can be used in peripheral tissues or converted to cholesterol and fatty acids. Low fecal SCFA levels might therefore exacerbate the cachectic metabolism, for instance, by failing to restrain excessive hepatic gluconeogenesis or by not providing adequate signaling to maintain insulin sensitivity [65]. However, since most of the intestinally produced SCFAs are filtered by the liver, the peripheral plasma levels of SCFAs are around the detection limit; thus, making it cumbersome to interpret these data.

Beyond SCFAs, other gut microbial metabolites and nutrient derivatives are altered in cachexia. One example is branched-chain amino acids (BCAAs). Certain gut bacteria consume dietary BCAAs, potentially reducing their availability for host muscle protein synthesis [25,29,31]. It has been documented that cachectic cancer patients (e.g., lung cancer) have lower plasma BCAA levels than non-cachectic patients [57], which could contribute to muscle wasting. This might be partly microbiota-driven: an overgrowth of BCAA-catabolizing bacteria could deplete these amino acids. Indeed, microbial dysbiosis in cachexia often features increases in organisms (like some *Bacteroidetes*) that metabolize branched amino acids [25,29,31]. Additionally, perturbations in bile acid profiles have been seen in cachexia as mentioned, cachectic mice had increased conjugated (host-phase) bile acids and reduced secondary (microbial) bile acids [53]. Since bile acids can function as metabolic regulators and signaling molecules (e.g., via FXR and TGR5 receptors), such changes might influence muscle and liver metabolism in cachexia [67].

Collectively, the metabolic changes in cachexia related to the microbiome include SCFA depletion, altered amino acid availability, and shifts in other gut microbial metabolites. These changes are likely interdependent with the inflammatory environment. For instance, low butyrate leads to a “leaky” gut, permitting LPS entry which triggers systemic cytokine release that further perturbs metabolism (causing insulin resistance, hyper catabolism). Thus, microbiome-induced metabolic derangements provide a mechanistic link between gut dysbiosis and the hallmark features of cachexia (hypermetabolism and muscle loss). Restoration of gut microbial metabolic outputs is being explored as a strategy to counteract these effects, as discussed in later sections.

### 2.3. Cancer Cachexia-Linked Dysbiosis May Lead to Pro-Inflammatory Cytokine-Mediated Skeletal Muscle and Adipose Tissue Wasting

Cancer cachexia is often accompanied by elevated circulating cytokines like IL-6 [68], IL-1β [69], and TNF-α [70] as well as acute-phase reactants (CRP, fibrinogen) in patients, resulting in a state of chronic systemic inflammation [71]. Importantly, these changes were not attributable solely to reduced food intake or malnutrition, as in experiments where control mice were pair-fed to match the lower intake of tumor-bearing cachectic mice, the controls did not develop the same degree of barrier dysfunction or dysbiosis [26]. The origin of this inflammation is multifactorial—besides the tumors’ role in inflammation [72], evidence also suggests that gut barrier dysfunction and its resulting microbial translocation may play a key role in amplifying inflammation [73]. Apc^Min/+^ mice (a model of colorectal cancer cachexia) demonstrating the concept of “leaky gut” showed increased permeability to large molecules compared to controls even before a significant weight loss phenotype [41]. The same study also implied that gut barrier dysfunction worsened with the increase of chronic IL-6, which may suggest that [41] a feed-forward loop exists where gut barrier loss and an increase in inflammation support each other. The gut barrier dysfunction can result in endotoxemia by increased LPS loads, thereby increasing LBP and pro-inflammatory cytokines, with severely cachectic Apc^Min/+^ mice showing five-fold higher endotoxin levels than non-cachectic mice [41]. Similarly, a C26 cachectic mouse showed significantly elevated serum LBP (an acute-phase protein that binds LPS) and pro-inflammatory cytokines, reflecting increased gut-derived LPS load [26]. These data indicate that the tumor and its inflammatory milieu (rather than starvation) are key drivers of gut barrier injury in cachexia.

Besides the cytokine IL-6 supporting a pro-inflammatory environment, producing LPS, and affecting gut barrier integrity [73], it also correlated to the upregulation of claudin-2 (a pore-forming tight junction protein) and the downregulation of sealing junction proteins (occludin, claudin-1), changes known to increase paracellular permeability [74]. In the human gastric cancer study by Jiang et al., cachectic patients had higher intestinal expression of claudins and lower occludin, consistent with a looser, more permeable epithelium [43]. These patients also had bacteria detectable in mesenteric lymph nodes (a sign of translocation) and elevated serum IL-6 and TNF-α levels [43]. Thus, in humans, as in mice, cachexia appears to be linked to a compromised gut barrier that lets endotoxins and microbes into systemic circulation, fueling inflammation. Fecal calprotectin levels, a marker of gut inflammation, have been found to be modestly increased in cachectic cancer patients and correlates with the abundance of certain bacteria (like Enterobacteriaceae and *Veillonella*) that thrive in inflammatory conditions [39]. This further supports the connection between gut microbial dysbiosis, local gut inflammation, and systemic inflammatory markers.

Systemic inflammation plays the central role in fueling the vicious cycle of cancer cachexia. This vicious cycle is maintained by individual feed-forward loops resulting in increased pro-inflammatory cytokines. Systemic inflammation has direct catabolic effects on skeletal muscle and adipose tissue, which explains why controlling inflammation can mitigate cachexia. Cytokines such as TNF-α and IL-6 have been shown to induce muscle wasting when administered chronically in animal models [71,72]. TNF-α activates the ubiquitin–proteasome pathway in muscle, accelerating protein degradation, and also impairs appetite and anabolic signaling (“cachectin” was an early name for TNF) [71,72]. IL-6, often elevated in cancer patients with cachexia, has been correlated with weight loss, reduced food intake, and depression; high IL-6 plasma levels are associated with shorter survival in cachectic patients [72]. Monoclonal antibodies that block IL-6 (e.g., tocilizumab, an IL-6 receptor (IL-6R) antagonist) have shown preliminary promise in attenuating muscle atrophy in cancer patients [75]. In the context of the microbiota–gut barrier axis, it is compelling that blocking IL-6 or IL-6R in cachectic mice not only reduced inflammation but also favorably altered the microbiota and improved gut integrity [76]. Treating C26 cachectic mice with anti-IL-6 antibody prevented the typical bloom of Enterobacteriaceae and gut leakiness seen in untreated cachectic mice, and those mice had less weight and muscle loss [76]. This suggests that IL-6 is a key mediator linking tumor and host to gut microbial changes. High IL-6 might drive dysbiosis and barrier damage, which then loops back to sustain IL-6 production, for instance, via LPS activation of monocytes [77]. Thus, targeting either side of this loop (inflammation or the gut) can break the cycle.

Another pertinent inflammatory mediator is LPS itself. The LPS endotoxin derived from Gram-negative bacteria, once in circulation, can directly activate muscle Toll-like receptors and lead to cytokine production and insulin resistance. LPS triggers muscle atrophy pathways by elevating pro-inflammatory cytokines (e.g., TNF-α, IL-1β, IL-6) that upregulate muscle-specific E3 ubiquitin ligases (atrogin-1/MafBx and MuRF1) which drive proteolysis [78,79,80]. In chronic infections and sepsis, gut barrier dysfunction has been correlated to muscle catabolism [81]. The same can be suggested for cancer cachexia; a recent study shows the leaky gut (often associated with cancer cachexia) mouse model leads to impaired systemic glucose metabolism and insulin tolerance in skeletal muscle which improved after soluble dietary fiber supplementation [82].

Analogous to what is seen in obesity (metabolic syndrome), cancer cachexia can also be described as a systemic metabolic syndrome where tumors drive the host’s hypermetabolic and insulin resistant state [9]. Gut barrier dysfunction plays a role in amplifying the cachexia state, but describing it as a state of “metabolic endotoxemia” might not be correct since the tumor has a primary and systemic effect [83]. In obesity studies, a high-fat diet caused gut microbiota changes and mild endotoxemia that led to systemic inflammation (termed “meta inflammation”) [83]. Taking the importance of tumor-induced effect, a parallel can still be drawn in cachexia: tumor factors induce dysbiosis and gut permeability, causing endotoxemia that drives muscle wasting and further inflammation. In support of this, broad-spectrum antibiotics in a non-cancer context (obese mice) have been shown to reduce circulating LPS and lower systemic TNF-α/IL-6 levels [42,84]. While cachexia involves more intense inflammation than obesity, these principles overlap.

In summary, cachexia’s inflammatory state is intertwined with intestinal health as gut microbiota perturbations and barrier defects expose the host to continuous immune stimulation from microbial products, exacerbating cytokine production. This chronic inflammation then feeds back negatively on the gut (for example, causing motility changes, mucosal atrophy, or further dysbiosis) and on peripheral tissues (causing anorexia, hypermetabolism, and muscle breakdown). The result is a self-perpetuating triad of dysbiosis, gut barrier dysfunction, and systemic inflammation. Figure 2a illustrates this cycle, and the data reviewed here provide concrete examples: *Klebsiella*-driven barrier leak in mice [40], IL-6-driven microbiota changes [26], and human evidence of endotoxin leak in cachexia [43]. Clinically, these insights highlight the importance of maintaining gut integrity in cancer patients. Showing promise in interventions that reduce gut permeability or inflammation might ameliorate cachexia progression.

### 2.4. Gut Microbiota-Targeted Therapies May Restore Eubiotic State, Thereby Mitigating Inflammation and Muscle Wasting

Given the association of gut dysbiosis and SCFA loss with cachexia, a logical next step is to test therapies that modulate the microbiota or its products to see if cancer cachexia outcomes can be improved. In recent years, several such strategies have been explored in preclinical models with encouraging results, and a few have entered clinical trials.

#### 2.4.1. Probiotics

Probiotics are live microorganisms, often lactate- and acetate-producing specific bacterial strains of *Lactobacillus* and *Bifidobacterium* or butyrate-producing *Clostridium butyricum* [85] that confer health benefits to the host. In the context of cancer cachexia, probiotics aim to rebalance the gut microbiota. This may lead to a more anti-inflammatory composition and strengthen the gut barrier. Bindels et al. provided one of the first proofs-of-concept by administering *Lactobacillus reuteri* to cachectic leukemia-bearing mice. Restoring this single *Lactobacillus* species, which was originally depleted in the leukemic mice, led to reduced systemic inflammation (lower plasma levels of IL-6) and decreased muscle atrophy markers compared to untreated cachectic controls [45]. This demonstrated that targeting a missing commensal could beneficially modulate the host’s inflammatory state and muscle metabolism. In another study, Varian et al. (2016)’s oral administration of *Lactobacillus reuteri* in mice with colon cancer cachexia increased gastrocnemius muscle mass and fiber size, lowered plasma TNF-α levels, and upregulated transcription factor FoxN1 (Forkhead Box N1) [58]. The latter is interesting, as FoxN1 is essential for thymopoiesis and T cell development, thereby mediating immune modulation and linking probiotic intake to preserved muscle mass via enhanced thymic function [58]. These findings suggest that replenishing specific gut bacteria can dampen the pro-cachectic inflammatory signals.

Probiotics may also exert effects by increasing SCFA production. Some strains of *Bifidobacterium* and *Lactobacillus* produce acetate or lactate that cross-feed butyrate-producing microbes. This helps establish stable and invasion-resistant gut microbiota [86,87,88,89]. Lachnospiraceae (butyrate producers) has the ability to grow in the presence of lactate and acetate with the net stoichiometry of 4 mols of lactate, and 2 mols of acetate, producing 3 mols of butyrate [90]. By maintaining eubiosis, probiotics may help sustain SCFA output, which in turn supports gut barrier integrity by acting as an energy substrate [91] and immune modulation by activation of G-protein-coupled receptors (GPCRs) like FGAR2 and FFAR3 [92], and inhibition of histone deacetylases (HDACs) [88,89,93,94]. However, a study by Bindels et al. was unsuccessful in restoring gut barrier function in the C26 cancer cachexia model using *F. prausnitzii* [26] which has previously been shown to improve gut barrier function in other murine models for colitis [95], low-grade inflammation [96], and non-inflammatory irritable bowel syndrome (IBS) [97]. It should be noted, however, that effective (next generation) probiotic strain therapy or use of synthetic bacterial strain consortia requires following the FAO and WHO criteria [98] requiring strains to survive gastric high acid and, thus, low pH in the stomach and, moreover, withstand the higher bile acid concentrations in the small intestine. Finally, engraftment of these probiotic bacterial strains that need to colonize or at least persist in the gut at adequate numbers (usually ≥10^7^ CFU) is also cumbersome [99,100]. Further, focusing specifically on the mycobiome of the human gut microbiome, LLC-CC fungal dysbiosis [54,55], Jabes et al. demonstrated that fungal dysbiosis can also affect the development of cancer cachexia in mice. And further suggested *Rhyzopus oryzae* (Mucoromycota) as a promising probiotic candidate aimed at treating cancer cachexia [56]. One thing to be considered in cachectic patients, the gut may be a harsher environment (due to factors like bile acid alterations or antibiotics used during chemotherapy), so strain selection and dosing are crucial considerations.

#### 2.4.2. Prebiotics and Synbiotics

Dietary prebiotics, as defined by the 6th Meeting of the International Scientific Association of Probiotics and Prebiotics (ISAPP) in 2008, are ingredients that may or may not be selectively [101] fermented by intestinal microbiota and are resistant to mammalian enzyme hydrolysis, stomach acidic pH, and not absorbed in the gastrointestinal tract [102]. In cancer cachexia models, prebiotics have been used to try to boost endogenous SCFA producers. Inulin-type fructans (ITFs) are soluble fibers with a degree of polymerization equal to or higher than three have shown promise. Bindels et al. showed that in a mice model of leukemia and cachexia (BaF), combining the prebiotic inulin with *L. reuteri* as a symbiotic (since *L. reuteri* cannot metabolize ITFs [103]), resulted in significant improvements in gut barrier function, restored Lactobacillus levels, but did not show any significant change in the metabolite levels compared to the non-treated mice [48]. The inulin provided fermentable substrate that increased populations of beneficial commensals, while the probiotic seeded specific Lactobacilli together, they led to improved gut barrier function (evidenced by reduced intestinal permeability and enhanced tight junction protein expression) [104]. The mice that received synbiotic treatment had reduced muscle wasting and even prolonged survival despite an ongoing tumor burden [48]. Similar results were seen in a previous study by Bindels et al. in using ITFs in the BaF3 liver cancer model, although not specifically cachexia, suggesting that propionate might be responsible for the anti-tumor effect, although they found no direct correlation to the anti-inflammatory effect [45]. Other studies, not specifically on cancer cachexia, did suggest that propionate has an anti-inflammatory effect in human monocytes and the colitis model [105,106]. Another study tested a resistant starch (a fermentable fiber) with encapsulated probiotics in a murine colorectal cancer cachexia model [107]. The combination significantly attenuated weight loss and muscle wasting, likely by increasing cecal butyrate production and counteracting 5-Fluorouracil (5-FU) chemotherapy-induced dysbiosis [107]. These examples underscore that dietary supplementation with specific fibers can reshape the microbiome in favor of SCFA-producing and anti-inflammatory flora. This effect is linked to mitigating cachexia symptoms in animals.

From a clinical perspective, prebiotics (like resistant starch, inulin, fructo-oligosaccharides) are attractive because they are non-living, easy to administer as supplements or functional foods, and generally safe. They could be given alongside existing nutritional support as usually given to cancer patients. One phase II clinical trial in humans involves grape seed flour (GSF), a fiber- and polyphenol-rich supplement, in colorectal cancer patients undergoing surgery, aiming to modulate gut bacteria and inflammation during the perioperative period [108]. The rationale is that GSF, as a prebiotic, may reduce oxidative stress and inflammation [109] (grape polyphenols can favor beneficial microbes and inhibit pathogens) [110,111,112].

#### 2.4.3. Fecal Microbiota Transplantation (FMT)

FMT involves transferring an entire community of gut microbes from a healthy donor to a patient. The aim is to reset the recipient’s dysbiotic microbiome to an eubiotic state. Usually, 25% of the fecal discharge is the dry mass, of which 55% constitutes microorganisms [113], consisting mainly of prokaryotes (ratio of 10 to 1) [114], with 49% of the microbes being dead [115]. Thus, FMT involves transferring the donor’s dry fecal discharge of live and dead microbiota (bacteria, fungi, archaea, and viruses, with 95% being phages) as well as soluble components (mucus, proteins, fat, small molecules like bile acids, metabolites, along with short-chain fatty acids and colonocytes) [116]. It has proven efficacy in conditions like *Clostridioides difficile* infection [117] and is being explored in metabolic [118] and inflammatory diseases. In cancer cachexia, the concept is to transplant a “healthier” microbiome that could reduce inflammation and possibly improve nutritional status. The first-in-human trial of FMT in cachexia was recently conducted in which cachectic patients with advanced gastroesophageal cancer were randomized to either allogeneic FMT from a healthy, overweight donor (selected to provide a microbiome from a metabolically favorable phenotype) or autologous FMT (placebo) [59].

The FMT was given prior to standard chemotherapy. As reviewed in Table 1, the FMT did not lead to significant improvements in cachexia symptoms or body weight over 12 weeks [59]. Like the placebo group, patients in the FMT treatment group also continued to lose weight and had similar appetite scores. However, interestingly, the donor FMT group had a higher disease control rate and a trend toward improved progression-free and overall survival, suggesting altering gut microbiota via FMT can improve the response to therapy [59], considering a significant shift in gut microbiome composition was seen after allogenic treatment [59]. The authors suggest that maybe mycobiota or virome (bacteriophage being the most abundant genomic material in the fecal matter) [119] profiling can potentially find a correlation to the outcome, taking into consideration the importance of two unstudied kingdoms. Also, a limitation of this study was that no fecal SCFA or cytokine profiles (considered potential biomarkers of CC) were determined and subsequently linked to disease outcome, satiety or cachexia phenotype. Moreover, it remains a possibility that giving just one donor FMT was not sufficient to overcome ongoing cachexia drivers, or that the window was too short to see body composition changes in terminal cancer patients. Future FMT studies may need to optimize donor selection (e.g., donors with high SCFA production capacity), daily dosing frequency including encapsulated donor FMT, or combine FMT with nutritional support.

Another ongoing investigation (clinical study ID: NCT05606523) related to FMT is a study where germ-free mice are colonized with fecal microbiota from cachectic vs. non-cachectic pancreatic cancer patients. The goal is to see if the cachectic microbiome can induce cachexia features in mice, establishing causality. This kind of model could also be used to screen potential microbiome therapeutics (e.g., FMT from healthy donors or specific cocktails to rescue the phenotype). Additionally, a phase I clinical trial is examining the safety of FMT capsules in pancreatic cancer patients (not necessarily focused on cachexia, but on improving immunotherapy efficacy [120,121].

FMT from donors with previously favorable disease outcomes into patients with poor prognosis resulted in a larger clinical benefit compared to healthy donors [122]. Attention to fecal bacteriophages is often neglected while considering FMT. A study by Ott et al. in a relatively small study group of five people with *Clostridium difficile* infection (CDI) shows that sterile donor stool (Fecal filtrate transplant [FFT]) is sufficient to treat CDI, suggesting the importance of bacteriophages along with bacterial components and metabolites [123]. FVT (fecal virome transplantation), by decreasing the risk of bacterial invasive infection, might be a safer option than FMT [124]. The FMT procedure is, in general, considered well tolerated and safe to administer to an immunocompromised recipient [125]. However, a case of a plausibly multi-resistant *E. coli* strain infection from the donor resulting in the death of two recipients [126] points to the importance of screening donor fecal matter for infectious diseases [127].

#### 2.4.4. Postbiotics

Postbiotics are bioactive compounds (non-live bacterial products) [128,129] produced through microbial metabolic activity (fermentation) [130], which directly or indirectly provide a beneficial effect on the host [131]. Postbiotics have an advantage over prebiotics, considering they pose no infection risk in immunocompromised individuals [132]. A study by Lahiri et al. focusing on muscle atrophy without cancer induction showed that treating germ-free mice with a mix of SCFAs containing sodium acetate, sodium butyrate, and sodium propionate increased skeletal muscle and decreased expression of *Atrogin-1* while increasing *Murf-1* expression [32]. A fungal postbiotics study showed Botryosphaeran (fungal (1 → 3)(1 → 6)-β-D-glucan produced by *Botryosphaeria rhodina MAMB-05*) administration resulted in decreased weight loss, increased lean muscle mass, and reduced tumor development compared to the control [133]. Postbiotics like SCFAs, mainly propionate and butyrate, have been correlated to cancer cachexia phenotype improvements of lowered systemic inflammation, improved gut barrier integrity, and reduced muscle wasting as shown in previous studies; although, to the best of our knowledge, no study using postbiotics as an intervention against cancer cachexia has been found so far. Studies of postbiotics individually on cancer or cachexia are interesting, but these findings cannot be extrapolated to a multifactorial disease like cancer cachexia.

#### 2.4.5. Antibiotics

While never a long-term solution due to the risk of antibiotic resistance of bacterial strains, oral antibiotics have been used experimentally to probe the role of microbiota in cachexia. Gram-negative bacteria are an exclusive source of LPS in the gut. For instance, in the cancer cachexia model, selective decontamination of Gram-negative bacteria may reduce LPS burden. Although, as shown in a randomized controlled trial focusing on colorectal cancer surgery patients (no information on cachexia-positive status), selective decontamination of Gram-negative bacteria also results in a reduction of other microbes, leading to a total shift in microbiota composition [134,135]. Thus, suggesting that broad spectrum antibiotics can also worsen dysbiosis by killing beneficial taxa. However, anticancer therapy can drive gut microbial pathogen strain overgrowth, resulting in life-threatening infections; hence, to prevent or treat this situation, antibiotics are often administered [136]. Sulfisoxazole is an antibiotic against a wide range of Gram-positive and Gram-negative bacteria with potential anti-tumor effect in breast cancer models [137] and has been shown to inhibit lipolysis in C26 cachexia mice without showing any significant inhibition against tumor progression or muscle atrophy [138]. A study in obese mice (analogous to “meta inflammation”) showed that a cocktail of non-absorbable antibiotics lowered systemic inflammation [84], but in cancer cachexia, this approach has not been systematically tested. One concern is that advanced cancer patients are often already prone to infections and on antibiotics; thus, further antibiotic use might have drawbacks like fungal overgrowth or resistance. However, short-term gut decontamination targeting specific pathobionts (e.g., an anti-Enterobacteriaceae strategy) could be explored in animal models to see if it alleviates cachexia. As an example, a study cited earlier found that colonizing cachectic mice with *Klebsiella oxytoca* worsened gut integrity [40], implying that eradicating such a microbe could be beneficial. Any antibiotic strategy would need to be balanced with potential negative effects on the microbiome.

#### 2.4.6. Dietary Strategies

Beyond adding isolated fibers (prebiotics), overall diet composition profoundly influences the gut microbiome and might thus affect cachexia trajectory. High-protein, high-calory diets are standard recommendations for cancer cachectic patients to mitigate weight loss, but the role of diet quality (fiber content, fatty acid composition) is less studied. A recent animal study examined a walnut-supplemented diet (rich in omega-3s and polyphenols) in rats with two different tumor types to see if it alters the gut microbiome and cachexia course [139]. While the diet did modulate the microbiome, it did not significantly slow cachexia progression in that study [139], perhaps due to the severity of tumor-driven effects. In contrast, a “high-fiber diet” clinical trial (in melanoma patients) suggested that patients consuming >20 g fiber/day had better responses to immunotherapy, potentially linked to a more diverse microbiome [140]. Extrapolating to cachexia, a high-fiber, plant-rich diet could help maintain microbial diversity and SCFA production, possibly tempering inflammation. However, late-stage cancer patients often cannot tolerate such diets due to anorexia or gastrointestinal dysfunction [141,142].

Another dietary approach is supplementation with omega-3 fatty acids (fish oil), which have anti-inflammatory effects and have been tested in cachexia (with mixed results on weight loss). Omega-3 can also influence the gut microbiome composition (promoting certain beneficial bacteria) [143]. Some trials combined omega-3 with probiotics or other nutrients. For example, an enriched nutritional drink containing eicosapentaenoic acid (EPA), high protein, and prebiotic fiber was tested in cachectic patients and found to improve some immune parameters [22]. In a murine model, a multi-nutrient formula (including oligosaccharides as prebiotics) attenuated cachexia development [48]. These multi-modal nutritional interventions likely work partly via modulating the microbiome and partly via direct anti-inflammatory or anabolic effects.

In summary, multiple gut microbiota-focused interventions show potential in cachexia management (Table 1 highlights several preclinical interventions and one clinical FMT trial). Probiotics, prebiotics, and synbiotics have consistently demonstrated reductions in inflammatory cytokines and muscle wasting in animal models [48]. FMT as a comprehensive reset has shown safety and some signals of efficacy in humans (improved clinical outcomes if not weight gain) [59]. Dietary fiber and bioactive nutrients are being integrated into clinical trials with the aim of modulating gut microbiota and inflammation [108].

However, considering cancer cachexia is a multi-organ and multifactorial syndrome, a multi-modal approach focusing on both preventing catabolism and favoring anabolism rather than a single drug is more likely to provide a satisfactory outcome [8], indicating the importance of gut microbiome intervention in mitigating symptom cluster of cancer cachexia. Figure 2b illustrates the role of microbiota-directed interventions in mitigating cancer cachexia-derived systemic inflammation.

## 3. Discussion

This review provides cross-sectional evidence (although no causality) that the gut microbiota undergoes significant changes in cancer cachexia. These changes are functionally intertwined with the syndrome’s hallmark pathways (inflammation and metabolism). Across different cancer types and experimental models, cachexia is generally associated with a shift toward a pro-inflammatory microbiota profile and a reduction in health-promoting microbes/metabolites. However, we also observed that microbiota alterations are not uniform; they can vary by tumor type and anatomical context.

In gastrointestinal cancers (such as colon and pancreatic tumors), cachexia-related dysbiosis often features loss of butyrate-producing Firmicutes and overgrowth of Proteobacteria like Enterobacteriaceae [40]. This pattern likely reflects local influences. GI tumors might cause malabsorption or altered gut secretions that directly perturb the microbiome. For instance, pancreatic cancer patients with exocrine insufficiency may have more undigested nutrients reaching the colon, favoring different bacterial blooms [144]. Extra-intestinal cancers (e.g., lung cancer or leukemia) also induce dysbiosis, but the signals driving it are systemic (cytokines, hormonal changes, etc.) rather than direct luminal effects [145]. The LLC lung cancer model showed an increase [49] in certain Firmicutes families rather than a decrease [44], suggesting that cachexia can manifest with different dysbiotic signatures. One hypothesis is that leukemia and colorectal cancer models share common inflammatory mediators (like IL-6) that selectively suppress some microbes and encourage others (e.g., high IL-6 may be toxic to strict anaerobes or alter bile acids to the detriment of Firmicutes), whereas the LLC model might involve additional factors (like tumor-derived catecholamines or other immune factors) that have a different impact on the microbiome. Despite these differences, a unifying theme is that gut microbial dysbiosis in cachexia often entails a loss of microbes that produce SCFAs and other nutrients and an increase in microbes that can trigger inflammation or have pathogenic traits.

The question of causality in the relationship between gut microbiota and cachexia is complex. Does gut dysbiosis actively drive cachexia progression, or is it merely a bystander effect of the cachectic state? Current evidence suggests a bidirectional relationship. On the one hand, cachexia (driven by the tumor and host immune response) clearly induces changes in the microbiome, e.g., IL-6 transgenic mice develop cachexia and concurrently exhibit altered gut microbiota, implying the host inflammatory environment shapes the microbiome [26,76]. On the other hand, experiments like FMT from cachectic hosts into germ-free mice will help determine if the microbiome alone can transmit cachexia features. Early hints of causality come from studies where modulation of the microbiome affected cachexia outcomes: the synbiotic treatment that prevented muscle wasting and prolonged survival in leukemic mice [48] suggests that the gut microbiota is not just a bystander, because actively changing it altered the course of cachexia. Likewise, *Klebsiella* administration worsens gut permeability in cachectic mice [40], demonstrating that a specific microbial change can exacerbate a cachexia-related phenotype (gut barrier dysfunction). The truth is likely that gut dysbiosis acts as an accelerant or amplifier of cachexia, rather than the primary spark. The initial trigger for cancer cachexia lies in tumor–host interactions (tumor-derived factors, systemic inflammation, neuro-hormonal changes). But once those triggers create a dysbiosis, leaky gut, a vicious cycle ensues where microbial products intensify inflammation and metabolic disturbances, thereby worsening cachexia in a self-perpetuating manner [24].

One interesting aspect is the gut–muscle axis dysfunction in cachexia. Healthy gut microbiota supports muscle maintenance through multiple mechanisms. These mechanisms include producing SCFAs and vitamins, modulating systemic levels of IGF-1, regulating bile acids, and even directly affecting muscle via microbial peptides that mimic host signaling. In cachexia, this axis appears disrupted. Low SCFAs and high endotoxin create an environment where muscle protein synthesis is blunted and breakdown is accelerated. There is emerging evidence that skeletal muscle in cachexia undergoes changes in gene expression related to inflammation (e.g., upregulation of TLRs and cytokine receptors) [80]. One can speculate that circulating microbial ligands (such as LPS or even peptidoglycan) might directly activate pattern recognition receptors in muscle, contributing to atrophy [146]. A recent concept in the field is “muscle–gut axis,” wherein muscle and gut communicate bidirectionally: muscle wasting can increase gut permeability (possibly via reduced physical activity affecting gut motility and microbe composition), and gut-derived inflammation can worsen muscle loss. This axis might explain why exercise, which can beneficially modulate the gut microbiome and strengthen the gut barrier, has some positive effects in cachectic patients, although often not enough to reverse cachexia fully [147].

This review also highlights the limitations and gaps in our current knowledge. First, much of the mechanistic insight comes from animal models, which, while valuable, may not fully capture the clinical scenario. Murine models often involve a single tumor type in young, inbred mice with controlled diets; a far cry from the heterogeneity of human cancer patients with different cancers, older age, variable diets, and medications like antibiotics or proton pump inhibitors that affect microbiota composition [148]. The human studies to date are mostly cross-sectional observations with relatively small sample sizes. The location of the microbial sample impacts microbial signatures and metabolites produced [149]. Additionally, humans possess a significantly smaller cecum [150], while mice do not have a designated large intestine as humans do, therefore it is important to be cautious when extrapolating data from findings [149]. These can show associations (e.g., cachectic patients have more Proteobacteria) but cannot prove cause or directionality. There is also potential confounding in human studies: cachectic patients often have more advanced cancer or are undergoing treatments that non-cachectic patients are not, and those factors themselves can alter the microbiome. For example, certain chemotherapies and radiation can induce mucositis and change gut flora composition [151]. Future studies should ideally compare cachectic vs. non-cachectic patients within the same cancer type and stage (to minimize tumor-specific effects) and account for treatments. In this regard, the suggestion by one study to examine cachexia in metastatic disease separately is apt [152].

Another gap is the paucity of longitudinal human data. We do not know if gut microbiota changes precede the onset of cachexia or are reciprocal in their actions. Does dysbiosis worsen as patients lose weight over time? Are there microbiome signatures that could predict which cancer patients will develop cachexia? Longitudinal profiling of patients from diagnosis through disease course, correlating microbiome dynamics with weight/muscle changes, would be extremely informative. Additionally, integrative “multi-omics” approaches (combining metagenomics, metabolomics, and host transcriptomics) like that of Pötgens et al. [40] can elucidate how microbial metabolites (SCFAs, BCAAs, bile acids, etc.) interact with host metabolism during cachexia. It is also noted that not all studies agree on specific bacterial changes; this inconsistency suggests that no single microbe is universally “the cachexia bug.” Instead, the overall community structure and functional output (like SCFA levels) might be more consistently relevant. It will be important to move beyond just cataloguing taxa to measuring microbial function in cachexia: e.g., assessing fecal SCFA output, gut permeability assays, and even measuring LPS levels in patient blood could be as important as sequencing the microbiome. In the clinical realm, a practical consideration is that collecting and analyzing stool microbiomes from very ill cachectic patients can be challenging (due to patient debility, irregular bowel movements, etc.), which might partially explain why data are limited. Despite these limitations, cumulative evidence strongly supports the microbiome as a contributing factor in cachexia pathology. The improvements seen in preclinical interventions indicate that the gut microbiome is a promising therapeutic target. If a simple probiotic or fiber supplement can reduce muscle wasting in mice [48], similar approaches might stabilize weight or inflammatory markers in human patients. Finally, the human donor FMT trial, while not improving weight, suggested positive systemic effects [59], hinting that tweaking the gut microbiome could improve patient outcomes in ways not immediately evident by weight change.

Another discussion point is the personalization of microbiome interventions. Cancer cachexia is not a one-size-fits-all condition; for example, a patient with cachexia in pancreatic cancer might have different microbiome issues (due to pancreatic enzyme insufficiency) than a patient with lung cancer cachexia. It is conceivable that microbiome-based therapies should be tailored: a pancreatic cancer patient might benefit from pancreatic enzyme replacement plus a different prebiotic mix than a lung cancer patient, who might need more focus on modulating immune-related microbes. Personalized nutrition plans based on a patient’s baseline gut microbiota are an area of active research in oncology. Additionally, genetic factors in the host, such as polymorphisms in innate immune receptors like TLRs, could influence how the microbiome interacts with the host and thus how cachexia develops [153]. These nuances will require more research to unravel but are worth considering as we strive for more targeted therapies.

Traditionally, cancer cachexia has been seen purely as a paraneoplastic metabolic syndrome. Integrating the microbiome into our understanding of cachexia may add a new dimension. It provides a link between the tumor, the gut, and peripheral tissues like muscle. This allows us to break the cycle of inflammation and muscle wasting from within the gut outward.

## Figures and Tables

**Table 1:** Key cross-sectional studies correlating changes in gut microbiome composition to cancer cachexia.

Study (Year)	Population/Model	Gut Microbiota Changes	SCFA Changes	Inflammation and Immune Markers	Cachexia and Metabolic Outcomes	Gut Barrier	Intervention and Outcome
Bindels et al., 2018 [26]	Colon cancer cachexia (C26) + parallel human cohort (152 colorectal and lung cancer patients with or without cachexia)	(Mouse) Enterobacteriaceae expansion, loss of butyrate-producers. (Human) Similar dysbiosis in cachectic vs. non-cachectic (b.i.)	(Mouse) ↓ Cecal acetate, butyrate. (Human) ↓ acetate (significant), overall ↓ SCFAs (trend) (b.i.)	(Mouse) ↑ inflammatory cytokines, acute-phase response. (Human) ↑ IL-6, ↑ LPS-binding protein (LBP) in cachexia (b.i.).	Cachexia severity correlated with gut barrier dysfunction and systemic inflammation.	Increased (mice) (b.i.)	Anti-IL-6 antibody in mice prevented dysbiosis and muscle wasting. No microbiota-targeted intervention in humans.
Sakakida et al., 2022 [37]	Colon cancer cachexia (C26) with high-fiber diet	↑ SCFA-producing taxa with fiber (e.g., Lachnospiraceae, *Bifidobacterium*)	Likely ↑ SCFAs (acetate, propionate, butyrate)	Fiber-fed cachectic mice: less muscle wasting, higher body/muscle mass. Suggests reduced systemic inflammation (IL-6, TNF-α not explicitly measured).	Partial rescue of cachexia without affecting tumor progression; improved metabolic/inflammatory status.	Not measured	Prebiotic fiber (partially hydrolyzed guar gum) increased muscle mass, body weight.
Pötgens et al., 2021 [38]	Colon cancer cachexia (C26)	↓ Diversity (↓ Lachnospiraceae, Ruminococcaceae, ↑ Enterobacteriaceae), altered fungal composition (mycobiome)	↓ Butyrate, ↓ acetate in cachectic mice	↑ IL-6, TNF-α, acute-phase proteins. Multi-omics: ↑ LPS biosynthesis, ↓ amino acid biosynthesis, altered bile acids, correlating with hepatic inflammation, muscle proteolysis.	Significant host–microbe metabolic disruption (↓ plasma amino acids, ↑ purine catabolism, etc.).	Increased (“leaky gut”)	Observational; highlights need to restore SCFAs, reduce endotoxin, rebalance bile acids.
Ubachs et al., 2021 [39]	Mixed cancers (pancreatic, breast, lung, ovarian); *n* = 107 (33 cachectic)	Cachexia: ↑ Proteobacteria (esp. Enterobacteriaceae), ↑ *Veillonella.* ↓ *Megamonas*, *Peptococcus* vs. non-cachectic	↓ Fecal acetate (significant), propionate/butyrate trended lower	High CRP, pro-inflammatory cytokines common in cachexia. ↑ fecal calprotectin correlated with Enterobacteriaceae, *Peptococcus* → colonic inflammation.	Bacterial richness similar across groups. Proteobacteria bloom linked to weight loss, systemic inflammation.	Likely increased	Observational; suggests targeting Proteobacteria overgrowth, restoring SCFAs.
Pötgens et al., 2018 [40]	Colon cancer cachexia (C26)	↓ Lachnospiraceae, Ruminococcaceae, Porphyromonadaceae families. ↑ Enterobacteriace-ae (*Klebsiella oxytoca*). Loss of butyrate-producers (b.i.)	↓ Butyrate, ↓ acetate (b.i.)	Cachexia: severe muscle wasting, ↑ IL-6. *Klebsiella* overgrowth increased inflammation.	Identified *Klebsiella* as a potential target; no deeper metabolic analysis here.	Increased (“leaky gut”) (b.i.)	Observational; probiotic (*Faecalibacterium prausnitzii*) did not improve barrier or cachexia.
Jiang et al., 2014 [43]	Gastric cancer: cachectic vs. non-cachectic patients	↓ Microbiota diversity in cachectic	Not reported	Cachexia group: ↑ gut permeability (sugar test), ↑ claudin, ↓ occludin, ↑ bacterial translocation, ↑ IL-6/TNF-α, CRP > 10 mg/L, >10% weight loss.	Demonstrates link: dysbiosis → leaky gut → systemic inflammation → cachexia.	Increased	Observational (no intervention). Reinforces link between dysbiosis (and likely reduced diversity), leaky gut, and systemic inflammation in human cachexia.
Bindels, Beck, et al., 2012 [45]	Murine acute leukemia (Ba/F3): cachectic vs. non-cachectic	↓ *Lactobacillus* spp. (*L. reuteri, L. gasseri*) Dysbiosis: ↓ Firmicutes, ↑ Proteobacteria (b.i.)	Not measured	Cachexia: ↑ muscle atrophy genes (Atrogin-1, MuRF1), ↑ IL-6, TNF-α, G-CSF, MCP-1, IL-4. Probiotic lowered these cytokines.	Severe muscle wasting, anorexia. Probiotic reduced IL-6 and muscle atrophy markers.	Not assessed	Probiotic (L. reuteri + L. gasseri) attenuated cachexia (↓ IL-6, ↓ muscle loss).
Bindels et al., 2016 [48]	Murine leukemia (Ba/F3) & colon cancer (C26)	↓ *Lactobacillus* spp., ↑ *Parabacteroides goldsteinii*, Enterobacteriace-ae. (b.i.) Synbiotic restored beneficial taxa	↓ C.ecal acetate, butyrate (C26 model) (b.i.)	Cachexia: ↑ IL-6. Synbiotic prolonged survival, indicating reduced inflammatory tone	Rapid muscle wasting, shortened survival. Synbiotic attenuated cachexia severity, improved lifespan.	Increased permeability	Synbiotic (inulin + L. reuteri) normalized gut microbiota, improved barrier, reduced muscle wasting.
De Maria et al., 2021 [49]	Lung cancer cachexia (Lewis lung carcinoma in mice)	↑ Diversity in cachexia. ↑ Firmicutes (Staphylococcaceae, Turicibacteraceae, Lachnospiraceae, Ruminococcaceae), ↑ minor phyla (Cyanobacteria, Tenericutes, TM7), ↓ Bacteroidetes	Not measured	Cachexia: severe muscle wasting. Likely ↑ IL-6, TNF-α (not explicitly shown). Possible immune shifts (Th17/Treg).	Suggests lung cancer cachexia differs from GI cancer models.	Not measured	Observational; indicates tumor-type–specific dysbiosis, no intervention tested.
Jeong et al., 2023 [50]	Lung cancer cachexia (Lewis lung carcinoma in mice)	↓ α-diversity in cachexia. ↑ *Bifidobacterium*, *Romboutsia*, ↓ *Streptococcus*	↓ Fecal acetate, ↓ butyrate	Marked muscle wasting. Likely ↑ IL-6 (not detailed). Loss of *Streptococcus* may disrupt cross-feeding.	Reinforces SCFA depletion as common in cachexia; specific taxa shifts vary by tumor type.	Not measured	Observational; highlights need for SCFA-boosting strategies to mitigate cachexia.
Ni et al., 2021 [57]	NSCLC patients (*n* = 31; 12 cachectic, 19 non-cachectic)	Cachexia: distinct β-diversity but no α-diversity loss. ↑ *Klebsiella oxytoca*, ↓ *Faecalibacterium prausnitzii*, *Prevotella copri*, *Lactobacillus* spp.	Overall ↓ SCFAs predicted (loss of multiple SCFA producers)	Cachexia: >5% weight loss. Microbiome shift: ↑ LPS/endotoxin synthesis, ↓ beneficial metabolic pathways (drives systemic inflammation, malnutrition). Branched-chain amino acids (BCAAs) significantly depleted.	“Pro-inflammatory, catabolic” microbiome profile.	Likely increased	Observational; suggests targeting *Klebsiella* and restoring SCFA-producers to mitigate cachexia. Non-cachetic patients: ↑ BCAAs ↑ 3-oxocholic acid showing positive correlation with *Prevotella copri* and *Lactobacillus gasseri*, respectively.
Varian et al., 2016 [58]	Colorectal cancer cachexia (Apc^Min/+^ mice)	Cachexia-associated dysbiosis (details not fully specified)	Not reported	Cachexia: severe muscle wasting. Probiotic group: ↑ muscle mass, ↓ atrophy, ↑ thymus size, ↓ FoxN1 (lower systemic inflammation).	Probiotic inhibited cachexia progression, prolonged survival.	Not reported	Probiotic (*Lactobacillus reuteri*) reduced muscle wasting, extended survival.
de Clercq et al., 2021 [59]	Metastatic gastroesophageal cancer, *n* = 24 (12 allogeneic FMT, 12 placebo)	Baseline dysbiosis in advanced cancer. Allogeneic FMT (healthy obese donors) transiently ↑ microbial diversity, ↑ SCFA-producers	Donors on high-fiber diets → likely ↑ SCFAs; recipient SCFAs not measured	No significant cachexia improvement (weight/appetite) with allogeneic vs. autologous FMT.	Allogeneic FMT group had higher disease control at 12 wks, trend of longer survival (365 vs. 227 days; *p* = 0.057), but weight loss persisted.	Not assessed	FMT did not reverse cachexia but improved chemo tolerance, hinted at survival benefit.
Bindels et al., 2015 [60]	Murine acute leukemia (cachexia), 2 weeks on 5% POS vs. control	↑ Bacteroidetes, *Bacteroides dorei*, *Bifidobacterium* spp., *Roseburia* spp. (a.i.)	↑ Acetate ↓ Isovalerate and other BCAA-derived SCFAs (a.i.)	Cachexia: anorexia, fat loss. Prebiotic reduced anorexia/adipose wasting (implying lower inflammation, though cytokines not shown).	Improved food intake, preserved fat mass with prebiotic (a.i.).	Not measured	Prebiotic (pectic oligosaccharides) improved appetite and reduced fat loss.
Castellani et al., 2017 [61]	Neuroblastoma cachexia (mouse model)	Minimal change vs. controls (slight ↓ Firmicutes, not significant)	No difference reported	Cachexia: ↑ IL-6, TNF-α. Altered gut hormones (↑ GLP-1, ↑ PYY). Marked muscle wasting despite stable microbiota.	↓ Secondary bile acids (e.g., lithocholic, deoxycholic), suggesting tumor-driven or inflammation-driven cachexia.	Not measured	No microbiota-targeted intervention (observational). Demonstrates cachexia can occur with minimal dysbiosis.
Hakozaki et al., 2022 [62]	NSCLC on immunotherapy (*n* = 113; 57 cachectic, 56 non-cachectic)	Cachexia: ↑ *Escherichia/Shigella*, *Hungatella*, ↓ *Anaerostipes*, *Blautia*.	Not measured, but fewer butyrate-producers → likely ↓ SCFAs	Cachexia: higher neutrophil–lymphocyte ratio, worse LIPI → severe systemic inflammation. Shorter survival under immunotherapy.	Loss of SCFA producers suggests lower anti-inflammatory metabolites (e.g., butyrate).	Not tested	Observational; *Anaerostipes* and *E. ventriosum* were associated with longer progression-free survival and overall survival.

(↑ = increased, ↓ = decreased, → = results in; b.i. = before intervention, a.i. = after intervention; MuRF1 = muscle ring finger protein 1, IL = interleukin, TNF = tumor necrosis factor, G-CSF = granulocyte colony stimulating factor, MCP-1 = monocyte chemoattractant protein-1, BCAAs = branched chain amino acids, SCFAs = short chain fatty acids, Ba/F3 = murine IL-3 dependent pro-B cell line, POS = pectic oligosaccharides, C26 = colon 25 cells, GLP-1 = glucagon-like peptide-1, PYY = peptide YY, LPS = lipopolysaccharide, FMT = fecal microbiota transplantation, Th17 = T helper 17, Treg = regulatory T cell, CRP = C-reactive protein, NSCLC = non-small cell lung cancer, LIPI = Lung immune prognostic index).

## Data Availability

No new data were created or analyzed in this study. Data sharing is not applicable to this article.
